# Burden of severe maternal peripartum mental disorders in low- and middle-income countries: a systematic review

**DOI:** 10.1007/s00737-021-01201-9

**Published:** 2022-01-21

**Authors:** Harish Kalra, Thach Tran, Lorena Romero, Prabha Chandra, Jane Fisher

**Affiliations:** 1 School of Public Health and Preventive Medicine, Monash University, Melbourne, Australia; 2 Ballarat Rural Clinical School, University of Notre Dame Australia, Sydney, Australia; 3 Grampians Area Mental Health Services, Ballarat, VIC Australia; 4 The Ian Potter Library, The Alfred Hospital, Melbourne, VIC Australia; 5 Department of Psychiatry, National Institute of Mental Health and Neurosciences, Bangalore, India

**Keywords:** Psychosis, Severe mental disorders, Peripartum, Low- and middle-income countries

## Abstract

Peripartum severe mental disorders (PSMDs) encompass schizophrenia, affective psychosis, and psychotic and non-psychotic forms of bipolar disorders. PSMDs are well documented in high-income countries. However, much less is known about the prevalence of PSMDs in low- and middle-income countries (LMICs). The aim was to review the available literature systematically and estimate the prevalence of PSMDs among women in LMICs. We searched the Ovid MEDLINE, Embase, PsycINFO, CINAHL and Maternity and Infant Care databases systematically from the date of inception to Dec 31, 2020, for English-language publications with data on the prevalence of PSMDs among women in World Bank–defined LMICs. Selection of studies, extraction of data and assessment of study quality were each undertaken independently by at least two of the investigators. A total of five studies (completed in three countries spanning two continents) met the inclusion criteria. Five studies reported cumulative incidence of postpartum psychosis (ranging from 1.1 to 16.7 per 1000 births). We found no studies on the prevalence of severe mental disorder during pregnancy in these settings. Marked heterogeneity in methodology precluded meta-analysis. These findings indicate that PSMDs occur at a similar prevalence in low- and middle-income to high-income countries. However overall, there is a paucity of high-quality evidence from these settings. There is a need for rigorous studies with standardized methods to increase knowledge of the nature, prevalence, and determinants of PSMDs among women in resource-constrained LMICs to inform policies, service development, program planning and health professional training.

**Supplementary Information:**

The online version contains supplementary material available at 10.1007/s00737-021-01201-9.

## Introduction

Mental health problems experienced by women who are pregnant or who have recently given birth (often referred to as the peripartum period) are common. These disorders contribute significantly to maternal morbidity and mortality, including obstetric complications, and adverse pregnancy outcomes and increase risk of self-harm and deaths by suicide (Howard and Khalifeh 2020). Peripartum mental disorders are broadly classified into two types: peripartum common mental disorders (PCMDs) and peripartum severe mental disorders (PSMDs) (Fisher et al. 2012; Jones et al. 2014). PCMDs constitute non-psychotic mental health problems including depression, anxiety, somatoform and adjustment disorders (Goldberg and Huxley 1992). PSMDs encompass schizophrenia, affective psychosis, and psychotic and non-psychotic forms of bipolar disorders (Jones et al. 2014).

PSMDs can be pre-existing with onset before or during pregnancy or after childbirth (Jones et al. 2014). Antepartum PSMDs are associated with negative obstetric and birth outcomes including preterm labour, pre-eclampsia, foetal distress, stillbirths, prematurity and foetal growth retardation (Howard et al. 2003; Vigod et al. 2014; Frayne et al. 2019; Heun-Johnson et al. 2019; Howard and Khalifeh 2020). PSMDs with onset in the immediate postpartum period are often collectively called postpartum or puerperal psychoses (Bergink et al. 2015). PSMDs in the postpartum period have a negative impact on mother-infant relationship (Brockington 2004; Gilden et al. 2020) and are associated with a higher risk of maternal deaths by suicide and infanticide (Brockington et al. 2017). Early identification and treatment are essential to minimise the risk of negative outcomes (Meltzer-Brody et al. 2018).

Prevalence studies of PSMDs have predominantly focussed on the postpartum period rather than pregnancy (Jones et al. 2014). The prevalence of postpartum psychosis is consistently estimated to be 1–2 per 1000 women who have recently given birth (Kendell et al. 1987; Perry et al. 2021). Establishing the true incidence and prevalence of an uncommon condition like postpartum psychosis is difficult, considering the relative rarity of the condition and the methodological challenges including need for assessment of very large samples and the logistical difficulties and costs associated with adequately sized prospective cohort studies. Most of the evidence about the prevalence of postpartum psychosis has been generated in World Bank–defined high-income countries (HIC) (Kendell et al. 1987; Terp and Mortensen 1998; Munk-Olsen et al. 2006; Vesga-López et al. 2008; Valdimarsdottir et al. 2009; Martin et al. 2016). A recent systematic review (VanderKruik et al. 2017) reported global incidence as being in the range of 0.89 to 2.6 per 1000 births and prevalence of 5 in 1000 births. However, this review only included studies with population samples of more than 200 women and having been published in the 15-year period (1990–2014). Most of the studies (4/6) were from HICs. Only two were from World Bank–defined low- and middle-income countries (LMICs).

In the first systematic review of the evidence, the prevalence rates of PCMDs were found to be much higher in LMICs than in HICs (Fisher et al. 2012). This finding has been consistently replicated in subsequent systematic reviews focussed on individual countries (Upadhyay et al. 2017; Duko et al. 2020; Kalra et al. 2021) regions (Mahendran et al. 2019; Prabhu et al. 2019; Dadi et al. 2020b, a; . 2020b, a; Endomba et al. 2021) and globally (Woody et al. 2017; Jha et al. 2018). However, this has not been explored in relation to PSMDs. Current evidence on PSMDs among women in LMICs is based on individual studies with no systematic synthesis or appraisal of the quality of the available evidence. The absence of systematic evidence around the burden of PSMDs in these settings may be indicative of failure to consider PSMDs in policy, programs and practices to address the needs of women with these severe and potentially disabling conditions in LMICs.

To fill this knowledge gap, the aims of this study were to review systematically the available evidence of prevalence of PSMDs experienced by women in LMICs, to provide summary prevalence estimates and to detect and delineate any difference, in prevalence of PSMDs among women in HIC.

## Methods

### Data source and search strategy

A multistep systematic search using the Preferred Reporting Items for Systematic Reviews and Meta-Analyses (PRISMA) guidelines (Moher et al. 2009) was conducted, in consultation with a specialist information analyst (LR). The protocol was registered prospectively with the international database of systematic reviews, PROSPERO http://www.crd.york.ac.uk/PROSPERO; ID = CRD 42,017,078,381.

A detailed literature search was performed in Ovid MEDLINE, Embase, PsycINFO, CINAHL and Maternity and Infant Care from the date of database inception to December 31, 2020, using a combination of Medical Subject Headings (MeSH) and text words (Box 1). We hand-searched the reference list of all included articles, to identify any relevant studies missed in the electronic searches. We did not search for unpublished, non-peer-reviewed grey literature.

### Study selection

Papers were eligible for inclusion if they:

- Reported empirical research done in at least one LMIC
- Were peer-reviewed and published in English
- Measured and reported prevalence data
- Included women who were aged at least 18 years who had been assessed using a clinical interview or a validated diagnostic instrument to establish whether their symptoms met diagnostic criteria for a PSMD prior to or during pregnancy or up to 1-year postpartum
- Reported data derived from cross-sectional, prospective or retrospective cohort or the baseline assessment from interventional studies

Review papers including meta-analyses, case reports and series, conference abstracts and posters were excluded.

### Study quality estimation

To evaluate study quality and risk of bias, the Standard Quality Assessment Criteria (SQAC) for Evaluating Primary Research Papers from a Variety of Fields (Kmet et al. 2004) were used. The items were scored 0 ‘Does not meet the criterion’, 1 ‘Partially meets the criterion’ and 2 ‘Meets the criterion’. We added a criterion for ‘Ethics approval’ with score of 0 for ‘No ethics approval’ and 1 ‘Has ethics approval’. An overall study quality score was calculated as the total score of all items divided by the total possible score, to yield a range from 0 (extremely low) to 1(extremely high quality). Items not applicable to the study were excluded from total possible score calculations.

### Data extraction

HK and TT independently screened the downloaded titles and abstracts against the inclusion criteria. Full texts of the papers meeting inclusion criteria and unclear citations were retrieved. HK and TT independently assessed the full text of the potentially eligible papers. HK, TT and JF reviewed and reached an agreement on the final papers included. HK, TT and JF each independently extracted data from all the included papers and assessed study quality. Decisions about extracted data and study quality were resolved by discussions and consensus among the investigators.

### Data analysis

A narrative synthesis and a meta-analysis subject to the availability and quality of the data.

## Results

**Fig. 1:**
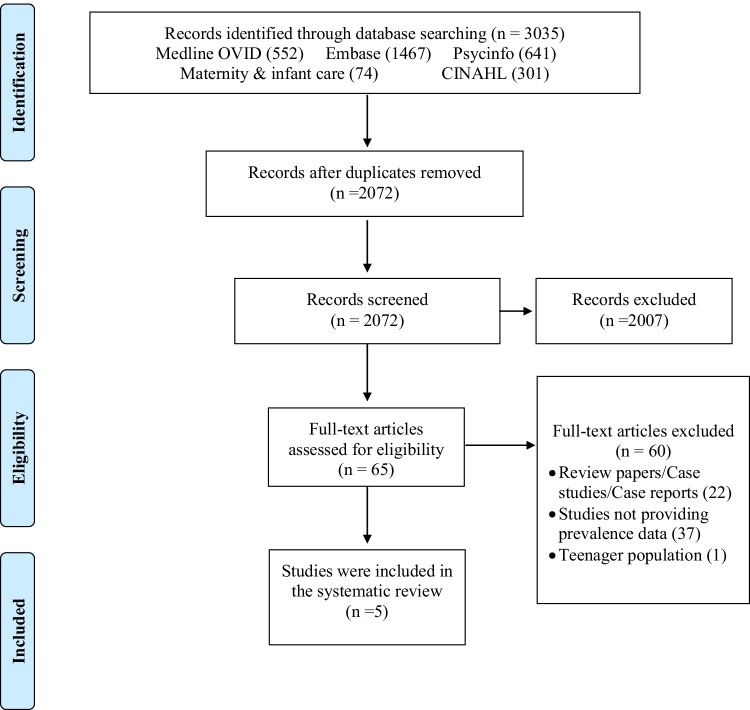
Flowchart showing the selection of studies for the systematic review of the prevalence of severe maternal peripartum mental disorders in LMICs

The PRISMA flow chart for the steps taken to select eligible papers based on the study criteria is displayed in Fig. 1. Five studies met criteria for inclusion in the review.

### Characteristics of the studies

The studies were conducted in Nigeria (*n* = 3), Tanzania (*n* = 1) and India (*n* = 1). All were published since 2000. Most (*n* = 4) were conducted in tertiary hospital inpatient settings. Only one study (Bang et al. 2004) recruited participants from a community-based population. In total these five studies reported on 167 women with a PSMD. Three studies (Ndosi and Mtawali 2002; Adefuye et al. 2008; Shehu and Yunusa 2015) calculated prevalence using the number of births in the hospital during the period of ascertainment as the denominator (162 women with a PSMD in a total of 69,055 births). The other two studies (Bang et al. 2004; Oyewole et al. 2014) used women who did not develop psychosis in the study populations as the denominator (5 women with a PSMD in a total of 952 women).

Two studies (Adefuye et al. 2008; Shehu and Yunusa 2015) extracted clinical information from audits of inpatient medical records to identify women diagnosed with postpartum psychosis retrospectively. Two studies (Ndosi and Mtawali 2002; Oyewole et al. 2014) were prospective and conducted within maternity settings. In Bang et al.’s study (2004), women were assessed repeatedly in their homes by community health workers until the 28th day postpartum. Only Oyewole et al. (2014) reported estimates based on locally validated outcome assessments.

All studies (*n* = 5) focussed on the postpartum period and reported cumulative incidence of new-onset PSMDs. There were variable definitions of the postpartum period, 28 days (Bang et al. 2004), 6 weeks (Ndosi and Mtawali 2002) and 8 weeks (Oyewole et al. 2014). Two studies (Adefuye et al. 2008; Shehu and Yunusa 2015) did not report periods of ascertainment. Further details of the studies are described in Table 1.

**Table 1:** Characteristics of the studies identified in the systematic review of the prevalence of severe maternal peripartum mental disorders in LMICs

	Postpartum				
	Shehu and Yunusa, 2015	Oyewole et al. 2014	Adefuye et al. 2008	Bang et al. 2004	Ndosi and Mtawali 2002
Country	Nigeria	Nigeria	Nigeria	India	Tanzania
Study setting	Tertiary hospital	Tertiary hospital	Tertiary hospital	Community	Tertiary hospital
Study type	Retrospective	Prospective	Retrospective	Prospective	Prospective
Perinatal period	Unclear	3 months	Unclear	4 weeks	6 weeks
	Postpartum	Postpartum only	Postpartum	postpartum	postpartum
Population and participants	Women diagnosed with postpartum psychosis after giving birth at the hospital in a 10-year period (2002–2011) Prevalence calculated as a fraction of all births at the hospital in the same period	Women diagnosed with postpartum psychosis after giving birth at the hospital in a 3-month period in 2003 Prevalence calculated as a fraction of women available for an initial interview within 48 h of the birth in the same period	Women diagnosed with postpartum psychosis after giving birth at the hospital in a 20-year period (1982–2007) Prevalence calculated as a fraction of all births at the hospital in the same period	Women who developed postpartum psychosis in a 12-month period (April 1995–March 1996) Prevalence calculated as a fraction of all women who delivered in 39 study villages in the same period	Women who developed postpartum psychosis after giving birth at the hospital in a 3-year period (1996–1998) Prevalence calculated as a fraction of all births at the hospital in the same period
Outcome	Postpartum psychosis	Psychiatric morbidity in the puerperium Prevalence calculated with postpartum psychosis data only	Postpartum psychosis	Maternal morbidity during labour and the puerperium Prevalence calculated with postpartum psychosis data only	Postpartum psychosis
Outcome measures	Medical records audit with ICD-10-based diagnosis	SCID-I GHQ-30	Medical records audit with the clinical diagnosis of postpartum psychosis	Unspecified	Research and diagnostic criteria for schizophrenia and affective disorders by Endicott and Spitzer ICD-10-based criteria
Validity/reliability of outcome indicators	Not reported	GHQ validity and cut-off reference provided	Not reported	Not reported	Not reported
Data collection procedure	Case notes of patients diagnosed with postpartum psychosis (ICD-10) in a 10-year period (Jan 2002 to Dec 2011) were retrieved manually by unspecified researchers from the health records Data relating to age, parity, presentation, risk factors, maternal and foetal morbidity/mortality were extracted and analysed for women with postpartum psychosis only	Initial psychiatric interview (within 48 h of birth) involved administration of sociodemographic questionnaire and GHQ-30 Follow-up interview (8 weeks postpartum) was conducted in postnatal/immunization clinic or during home visits GHQ-30 was administered on all follow up interviews (88.7%) Women scoring > 4 on the GHQ were further assessed with SCID-I to determine specific psychiatric disorders along with clinical interviews for the assessment of psychosocial factors	Case notes of patients diagnosed clinically with postpartum mental disorders in a 20-year period were extracted and analysed	Women were seen during 7th, 8th and 9th month of pregnancy by trained village health workers followed by 7 visits in the postpartum period (up to 28th day postpartum) to collect information by inquiry and/or observation for physical morbidity and abnormal speech or behaviour Quality of data collected was checked by a physician who also collected parallel forms independently on 119 consecutive mothers and neonates during his fortnightly visits to every village and therein, every house with a delivery Information on 18 variables related to maternal morbidities was compared to estimate the agreement between village health workers and the physician (95% agreement)	Clinical notes and additional information from the attending doctors Formal psychiatric interview was completed along with corroborative information from ‘relevant’ sources for a working psychiatric diagnosis
Number of cases/Sample size or *total number of births*	29/*25950*	3/180	23/*9085*	2/*772*	110/*34620*
Prevalence	1.1 per 1000 births	16.7 per 1000 births	2.5 per 1000 births	3 per 1000 births	3.2 per 1000 births
Study quality	0.62	0.62	0.52	0.76	0.62

*CIDI* Composite International Diagnostic Inventory, *ICD* International Classification of Mental and Behavioural Disorders, *SCID-I* Structured Clinical Interview for DSM- IV Axis I disorders, *GHQ* General Hospital Questionnaire

### Study quality

Individual detailed study quality scores on different dimensions of SQAC are presented in supplementary Table 1. Most studies consistently scored low on study design, sample selection and assessment measures. Analytic methods were not clearly described, and variance estimates were not reported. Only three out of five studies had approval from a formally constituted human research ethics committee. Two studies (Ndosi and Mtawali 2002; Adefuye et al. 2008) did not provide any information in relation to research ethics.

### Prevalence of PSMDs

The reported cumulative incidence of PSMDs among the included studies ranged from 1.1 to 16.7 per 1000 births. The highest prevalence estimate was reported by the study with the smallest sample (Oyewole et al. 2014). Cumulative incidence estimates in the other studies (Ndosi and Mtawali 2002; Bang et al. 2004; Adefuye et al. 2008; Shehu and Yunusa 2015) ranged from 1.1 to 3.2 per 1000 births.

Due to the small number of studies and marked heterogeneity in methods, we were unable to pool the data to calculate a summary estimate or conduct a meta-analysis.

## Discussion

To our knowledge, this is the first systematic review of the evidence available about the prevalence of PSMDs among women living in LMICs. Only five studies met the inclusion criteria.

### Prevalence of PSMDs

All studies investigated new-onset severe mental disorders in the postpartum period. Overall, the cumulative incidence of postpartum psychosis in the studies (4/5) included in this review (1.1 to 3.2 per 1000 births) is similar to the commonly reported population incidence (1–2/1000 births) (Kendell et al. 1987) and the findings of a recent global systematic review (0.89 to 2.6 per 1000 births) (VanderKruik et al. 2017) of the prevalence of postpartum psychoses globally.

### Methodological qualities of the included studies

There was substantial variation among studies in relation to sites of recruitment; sampling strategies; assessment measures, whether these had been locally validated; and definitions for case identification, which might have influenced the prevalence estimates. The varied denominators used in the included studies are likely to have influenced prevalence estimates. In all the settings in which these studies were completed, a proportion of women give birth at home. However, there is only one community prevalence study (Bang et al. 2004) in which women in this situation were included. Exclusion of women with mild or undetected PSMDs or those not requiring hospitalization or managed in community healthcare settings may have contributed to lower prevalence estimates. Misdiagnosis as psychosis, of organic states of delirium associated with infection or with nutritional deficits, may have led to overestimates of prevalence in Ndosi and Mtawali (2002)’s study (Brockington 2007). The finding of Oyewole et al. (2014) which is generalised from a relatively small sample appears to be an outlier. Their finding of high incidence rates (16.7 per 1000 births) of postpartum psychosis might be anomalous and less likely to be replicable, because they were generalised from a relatively small sample of 180 women. The likelihood of being able to establish reliable prevalence estimates of rare disorders like postpartum psychosis with such a small sample is extremely low (Arya et al 2012; Button et al 2013).

Current evidence is available for a relatively small number of women who gave birth at single hospitals. All were completed since 2000. Our findings are based on data from only 4 of more than 100 LMICs. Only one study from India and none from China met inclusion criteria. This is despite there being a high number of births in these two most populous LMICs. Moreover, multiple millions of women have given birth in LMICs and the accuracy of representation of population diversity cannot be assured. Together these limit capacities to form international estimates with certainty, but it is notable that in all countries in which there have been enquiries, women experiencing severe postpartum mental disorders have been found.

### Comparison with high-income countries

Despite the quite low quality of evidence, the incidence rates of postpartum psychosis among women in LMICs are near identical to those among women in high-income countries. This might be indicative of the primary role of biological underpinnings of these illnesses with potential combination of hormonal, immunological, circadian and genetic factors (Jones et al. 2014; Jones 2020) that might not vary among women worldwide. There was no evidence available about the prevalence of severe mental disorders (SMDs) including schizophrenia and bipolar disorder during pregnancy in LMICs, consistent with the relatively limited global literature on antepartum severe mental disorders (Jones et al. 2014). We found no studies of peripartum course and outcomes among women with a pre-existing PSMD in these settings.

The very small set of studies in this field in LMICs reflects the inherent difficulties in conducting epidemiological research about low prevalence disorders like PSMDs. However, it is also attributable to limited health services and research infrastructure and the lack of routine collection and reporting of mental health outcomes in maternity settings in LMICs. This is partially due to the high priority placed on reducing maternal mortality, and the physical conditions to which it is attributed. Mental health is not generally included in maternal health policy and programmes in these countries. The relative absence of resources such as vital registration systems including maternal mortality data, and population health data about maternal morbidities in these settings makes it challenging to determine the nature and extent of PSMDs in the resource-constrained settings of LMICs. This is in contrast to recent improvement in our understanding of the peripartum needs of women with PSMDs in HIC with use of these measures (Taylor et al. 2020) and regular confidential enquiries into maternal and peripartum deaths (Kurinczuk et al. 2014).

### Implications

Despite the low incidence rates, the absolute numbers of women with postpartum psychosis are alarmingly high. For example, there at least 25 million births annually in India alone, so absolute case numbers can be estimated at 25,000–50,000 per year (UNICEF, 2012). Psychosocial disadvantage and gender-related risk factors generally experienced by women in LMICs have a negative influence on help-seeking behaviours and reduce their access to treatment settings and resources. This potentially leads to amplified impact on the women, children and society in general with far more negative consequences compared to HIC settings. These factors make postpartum psychosis a public health priority, even in LMICs settings.

We could find no specific peripartum mental health assessment and treatment guidelines in resource-constrained LMICs. Our findings, in combination with high prevalence of PCMDs (Fisher et al. 2012; Woody et al. 2017; Jha et al. 2018), strongly support integration of mental healthcare into routine maternity care in these settings. This would improve women’s access to care and assist in the generation of country-specific data about the disease burden of peripartum mental health conditions and related risk factors for early child development (WHO 2018). Integrated maternity and mental healthcare would also provide an opportunity for delivery and scale up of maternal mental health interventions within existing mother and child health programmes, consistent with the World Health Organization mhGAP policy recommendations (WHO 2016).

### Strengths and limitations of the review

The major strengths of the review include prospective registration of the study protocol, comprehensive search strategy designed in collaboration with a specialist information analyst in five relevant databases and use of standardized data extraction and quality assessment protocols. We nevertheless acknowledge some limitations. We included only peer-reviewed published journal articles in English or with English-language abstracts. It is possible that we may have missed peer-reviewed papers or grey literature published in local languages.

## Conclusion

This evidence indicates that the prevalence of postpartum psychoses in LMICs is similar to that in HIC and therefore that all nations can estimate the need for appropriate mother-baby psychiatric services. The findings also highlight current knowledge gaps with the need for further quality research to calculate population prevalence estimates of PSMDs including postpartum psychosis among women in LMICs. This might be an opportunity for collaboration between researchers across countries in the Global North and the Global South with exchange of ideas and resources to help improve our understanding of these rare disorders. This will certainly help LMICs develop local evidence to inform policy development with not only inclusion of maternal mental health in the maternity care but also developmental of local intervention programs with improvement in maternal and child outcomes in LMICs.

## Supplementary Information

Below is the link to the electronic supplementary material.

Supplementary file1 (22.0 KB)

Supplementary file2 (21.7 KB)
