# Supplementary material for: Burden of severe maternal peripartum mental disorders in low- and middle-income countries: a systematic review
Source: Arch Womens Ment Health. 2022 Jan 21;25(2):267–75. doi: 10.1007/s00737-021-01201-9 (PMC8921056; doi:10.1007/s00737-021-01201-9)
Supplement: Supplementary file 1 — Supplementary file1 (22.0 KB) [file 737_2021_1201_MOESM1_ESM.docx]

**Supplementary Table 1.** **Methodological quality of studies of severe maternal peripartum mental disorders in LMICs**

| Criteria | Shehu & Yunusa, 2015^2^ | Oyewole et al 2014^3^ | Adefuye et al 2008^4^ | Bang et al 2004^5^ | Ndosi & Mtawali 2002^6^ |
| --- | --- | --- | --- | --- | --- |
| Objective | 1 | 2 | 2 | 2 | 2 |
| Study Design | 1 | 1 | 1 | 2 | 1 |
| Method of subject selection | 1 | 1 | 1 | 2 | 1 |
| Subject characteristics | 2 | 2 | 1 | 2 | 2 |
| If interventional, Random allocation | NA | NA | NA | NA | NA |
| If interventional, investigator blinding | NA | NA | NA | NA | NA |
| If interventional, subject blinding | NA | NA | NA | NA | NA |
| Outcome well defined | 1 | 1 | 1 | 0 | 1 |
| sample size | 2 | 1 | 2 | 1 | 2 |
| Analytic Methods | 1 | 1 | 1 | 2 | 1 |
| Variance estimates | 0 | 1 | 0 | 1 | 0 |
| Confounding controlled | NA | NA | NA | NA | NA |
| Results in sufficient detail | 1 | 1 | 1 | 2 | 2 |
| Conclusions supported by results | 2 | 1 | 1 | 2 | 1 |
| Ethics Approval | 1 | 1 | 0 | 1 | 0 |
| Total Possible score | 21 | 21 | 21 | 21 | 21 |
| Final Score | 13 | 13 | 11 | 16 | 13 |
| Study Quality Score | 0.62 | 0.62 | 0.52 | 0.76 | 0.62 |

**References**

1. Shehu CE, Yunusa MA. Obstetric Characteristics and Management of Patients with Postpartum Psychosis in a Tertiary Hospital Setting. *Obstet Gynecol Int*. 2015; **2015**.
2. Oyewole AO, Adelufosi AO, Abayomi O. Prevalence and correlates of puerperal psychiatric morbidity among attendees of a tertiary hospital in Northern Nigeria. *International Journal of Medical Science and Public Health*. 2014; **3**: 1402-7.
3. Adefuye P, Fakoya T, Odusoga O, Adefuye B, Ogunsemi S, Akindele R. Post-partum mental disorders in Sagamu. *East Afr Med J*. 2008; **85**: 607-11.
4. Bang RA, Bang AT, Reddy MH, Deshmukh MD, Baitule SB, Filippi V. Maternal morbidity during labour and the puerperium in rural homes and the need for medical attention: A prospective observational study in Gadchiroli, India. *Bjog-Int J Obstet Gy*. 2004; **111**: 231-8.
5. Ndosi NK, Mtawali MLW. The Nature of Puerperal Psychosis at Muhimbili National Hospital: Its Physical Co-Morbidity, Associated Main Obstetric and Social Factors. *African Journal of Reproductive Health / La Revue Africaine de la Santé Reproductive*. 2002; **6**: 41-9.
