# Supplementary material for: Burden of severe maternal peripartum mental disorders in low- and middle-income countries: a systematic review
Source: Arch Womens Ment Health. 2022 Jan 21;25(2):267–75. doi: 10.1007/s00737-021-01201-9 (PMC8921056; doi:10.1007/s00737-021-01201-9)
Supplement: Supplementary file 2 — Supplementary file2 (21.7 KB) [file 737_2021_1201_MOESM2_ESM.docx]

**Supplementary Box 1. Search strategy for systematic review of the evidence on the prevalence of severe maternal peripartum mental disorders in LMICs**

| **#** | **Searches** |
| --- | --- |
| 1 | exp Pregnancy/ |
| 2 | (pregnan* or childbirth or parturition* or gestation* or antenatal or prenatal or perinatal or postnatal or natal or gravidit* or gravida* or multigravid* or primigravid*).mp. |
| 3 | (postpartum or prepartum or peripartum or antepartum or intrapartum or partum or parity or parous or primiparity or multiparity or primiparous or multiparous or puerper*).mp. |
| 4 | (expectant adj (mother* or wom#n or female*)).mp. |
| 5 | 1 or 2 or 3 or 4  **Concept A = Perinatal setting (this line contains the group of terms for the Perinatal setting)** |
| 6 | exp "schizophrenia spectrum and other psychotic disorders"/ or psychotic disorders/ or affective disorders, psychotic/ |
| 7 | (psychos?s or psychotic* or delusional* or schizo*).mp. |
| 8 | (severe adj (mental* or psychiatr* or delusion* or hallucinat*)).mp. |
| 9 | 6 or 7 or 8  **Concept C = Mental Disorders (this line contains the group of terms for the Mental disorders)** |
| 10 | Developing Countries/ |
| 11 | (Afghanistan* or Albania* or Algeria* or Angola* or Argentina* or Armenia* or Azerbaijan* or Bangladesh* or Belarus* or Beliz* or Benin* or Bhutan* or Bolivia* or Bosnia* or Herzegovin* or Botswan* or Brazil* or Bulgaria* or Burkina* or Burundi* or Cabo Verde* or Cape Verde* or Cambodia* or Cameroon* or Central African or Chad* or China or Chinese or Colombia* or Comor* or Congo* or Costa Rica* or Cote d'Ivoir* or Ivory Coast or Cuba* or Djibouti* or Dominica* or Ecuador* or Egypt* or El Salvador* or Eritrea* or Ethiopia* or Fiji* or Gabon* or Gambia* or Georgia* or Ghana* or Grenad* or Guatemala* or Guinea* or Guyan* or Haiti* or Hondura* or Hungar* or India* or Indonesia* or Iran* or Iraq* or Jamaica* or Jordan* or Kazakhstan* or Kenya* or Kiribati* or Korea* or Kosov* or Kyrgyz Republic or Lao* or Leban* or Lesotho* or Liberia* or Libya* or Macedonia* or Madagascar* or Malawi* or Malaysia* or Maldiv* or Mali* or Marshall Island* or Mauritania* or Mauriti* or Mexic* or Micronesia* or Moldova* or Mongolia* or Montenegr* or Morocc* or Mozambi* or Myanma* or Burmese or Namibia* or Nepal* or Nicaragua* or Niger* or Nigeria* or Pakistan* or Palau* or Panama* or Papua New Guinea* or Paraguay* or Peru* or Philippines or Filipino or Romania* or Rwanda* or Samoa* or Sao Tome* or Senegal* or Serbia* or Seychell* or Sierra Leon* or Solomon Island* or Somalia* or South Africa* or Sudan* or Sri Lanka* or St Lucia* or St Vincent or Grenadines or Surinam* or Swazi* or Syria* or Tajikistan* or Tanzania* or Thai* or Timor* or Togo* or Tonga* or Tunisia* or Turk* or Turkmenistan* or Tuvalu* or Uganda* or Ukrain* or Uzbekistan* or Vanuatu* or Venezuela* or Vietnam* or West Bank or Gaza or Yemen* or Zambia* or Zimbabwe*).mp. |
| 12 | exp africa/ or exp caribbean region/ or exp central america/ or latin america/ or exp south america/ or asia/ or exp asia, central/ or exp asia, southeastern/ or exp asia, western/ or exp indian ocean islands/ or pacific islands/ or exp melanesia/ or exp micronesia/ or exp west indies/ |
| 13 | (africa* or asia* or caribbean or central america* or latin america* or south america* or melanesia* or micronesia* or polynesia*).mp. |
| 14 | (resource-limit* or resource-poor or low-resource* or limited-resource* or resource-constrain* or constrain*-resource* or under-resource* or poor*-resource* or resource-scarce* or scarce*-resource* or low-income or middle-income or lowincome or middleincome or (low adj3 middle-income)).mp. |
| 15 | ((developing or underdeveloped or under-developed or emerging or less-developed or least-developed or less-economically developed or least-economically developed or less-affluent or least-affluent or deprived or poor) adj (country or countries or nation or nations or region or regions or economy or economies)).mp. |
| 16 | ((developing or underdeveloped or under-developed or less-developed or least-developed) adj (population* or world)).mp. |
| 17 | (third-world* or thirdworld* or 3rd-world* or lmic or lmics or lami countr* or lalmi countr* or transitional countr*).mp. |
| 18 | (low* adj (gdp or gnp or gross domestic or gross national)).mp. |
| 19 | 10 or 11 or 12 or 13 or 14 or 15 or 16 or 17 or 18  **Concept C = LMIC (this line contains the group of terms for low- and middle-income countries** |
| 20 | 5 and 9 and 19  **This line combines A) Perinatal setting and B) Mental Disorders and C) LMICs** |
| 21 | limit 20 to english language |
| 22 | exp animals/ not humans.sh. |
| 23 | 21 not 22  **This line provides the result of A and B and C in the English language but without animal studies** |
